# Community predictors of COVID‐19 cases and deaths in Massachusetts: Evaluating changes over time using geospatially refined data

**DOI:** 10.1111/irv.12926

**Published:** 2021-11-10

**Authors:** Keith R. Spangler, Prasad Patil, Xiaojing Peng, Jonathan I. Levy, Kevin J. Lane, Koen F. Tieskens, Fei Carnes, R. Monina Klevens, Elizabeth A. Erdman, T. Scott Troppy, M. Patricia Fabian, Jessica H. Leibler

**Affiliations:** ^1^ Department of Environmental Health Boston University School of Public Health Boston Massachusetts USA; ^2^ Department of Biostatistics Boston University School of Public Health Boston Massachusetts USA; ^3^ Bureau of Infectious Disease and Laboratory Sciences Massachusetts Department of Public Health Boston Massachusetts USA; ^4^ Office of Population Health Massachusetts Department of Public Health Boston Massachusetts USA

**Keywords:** census tract, COVID‐19, disparities, geocoding, race/ethnicity, SARS‐CoV‐2

## Abstract

**Background:**

The COVID‐19 pandemic has highlighted the need for targeted local interventions given substantial heterogeneity within cities and counties. Publicly available case data are typically aggregated to the city or county level to protect patient privacy, but more granular data are necessary to identify and act upon community‐level risk factors that can change over time.

**Methods:**

Individual COVID‐19 case and mortality data from Massachusetts were geocoded to residential addresses and aggregated into two time periods: “Phase 1” (March–June 2020) and “Phase 2” (September 2020 to February 2021). Institutional cases associated with long‐term care facilities, prisons, or homeless shelters were identified using address data and modeled separately. Census tract sociodemographic and occupational predictors were drawn from the 2015–2019 American Community Survey. We used mixed‐effects negative binomial regression to estimate incidence rate ratios (IRRs), accounting for town‐level spatial autocorrelation.

**Results:**

Case incidence was elevated in census tracts with higher proportions of Black and Latinx residents, with larger associations in Phase 1 than Phase 2. Case incidence associated with proportion of essential workers was similarly elevated in both Phases. Mortality IRRs had differing patterns from case IRRs, decreasing less substantially between Phases for Black and Latinx populations and increasing between Phases for proportion of essential workers. Mortality models excluding institutional cases yielded stronger associations for age, race/ethnicity, and essential worker status.

**Conclusions:**

Geocoded home address data can allow for nuanced analyses of community disease patterns, identification of high‐risk subgroups, and exclusion of institutional cases to comprehensively reflect community risk.

## 1 INTRODUCTION

The COVID‐19 pandemic has exacerbated existing racial and ethnic health and socioeconomic disparities in the United States. Notably, Black/African American, Latinx, and Indigenous populations have suffered disproportionate morbidity and mortality,^1^, ^2^, ^3^, ^4^, ^5^ as well as financial loss from subsequent economic disruption.^6^ These disparities are substantially due to systemic racism and its consequences that affect infectious disease transmission and recovery, including unequal access to medical care,^7^, ^8^ suboptimal housing characteristics,^9^, ^10^ and employment in essential services with minimal physical distancing.^11^ While the literature highlights the significant burden on communities of color in the United States as a result of the pandemic, there are few analyses to date that evaluate these disparities at higher resolution than the county level, and even fewer that disentangle cases originating in institutional congregate settings. Using stratified, higher‐resolution data, we may be able to identify important community‐level conditions that contribute to the clear and persistent disparities induced by the pandemic.

In particular, it has been widely recognized that older Americans, especially individuals living in nursing homes or assisted‐living facilities, have faced significantly elevated risk of COVID‐19 morbidity and mortality, especially early in the pandemic.^12^, ^13^, ^14^ Similarly, high case burdens have been observed in other institutional residential settings, such as prisons and homeless shelters.^15^, ^16^ Models using total case and mortality rates without removing or controlling for these institutional settings may obfuscate trends or risk factors in community transmission. Since the racial and ethnic composition of institutional and non‐institutional settings may differ, disparities may be better characterized using higher‐resolution data. In addition, characteristics and risk factors associated with disease transmission and severity within institutional settings may not coincide with those driving COVID‐19 transmission within non‐institutional community settings.^17^ Efforts to disaggregate institutional and community outcomes would inform a more comprehensive understanding of health disparities in both institutional and non‐institutional settings, in turn directing testing efforts and informing mitigation activities.

Analyses using highly resolved geospatial data provide the tools to identify specific, local factors that contribute to disease outcomes and hone targeted efforts to intervene and support communities. Such data are particularly useful for local public health departments, for whom local data from their community is more valuable than aggregated larger‐scale trends. Local case data coupled with community‐level sociodemographic data at the census‐tract level can provide public health leaders with actionable and highly relevant local information and support pandemic response.^18^, ^19^, ^20^, ^21^

State public health departments have served a critical role during the pandemic in collecting and aggregating individual patient information across municipalities within the state and communicating relevant information back to local leaders. Due to patient privacy regulations,^22^ data on residential addresses associated with COVID‐19 that would support generating census tract resolution case estimates and distinguishing institutional from non‐institutional cases are not publicly available. Cross‐sectoral partnerships and data sharing agreements between state public health agencies and academic researchers can support analyses that integrate protected public health data with community‐level characteristics. This study reflects one such partnership between the Massachusetts Department of Public Health (MDPH) and academic researchers at Boston University School of Public Health to inform state and local interventions to mitigate COVID‐19 risk and associated health disparities.

In this study, we geocoded residential home addresses (street, city/town, and zip code) of individual COVID‐19 cases confirmed via nucleic acid amplification tests (NAAT) from the first year of the COVID‐19 pandemic in Massachusetts (MA), as provided by MDPH. We then analyzed community‐level sociodemographic and occupational predictors of outcomes at the census‐tract level. The goals of this project were to (1) estimate associations between community‐level risk factors and COVID‐19 cases and deaths by census tract in the state; (2) evaluate the sensitivity of these associations to the exclusion of institutional cases and deaths, given the relevance of institutional settings to larger‐scale disease patterns; and (3) assess changes over time in these associations during the initial two periods of high case burdens in the state.

## 2 METHODS

### 2.1 Data sources

Individual‐level COVID‐19 case and mortality data from March 2020–February 2021, including residential address and date of diagnosis or death, were provided by MDPH. Residential addresses were geocoded using a cascade matching geocoding method developed by combining MassGIS Address Points for Geocoding^23^ and ArcGIS Business Analyst geocoding locators (© Esri, Redlands, CA), and assigned to the census tract associated with home locations (1462 tracts within MA after dropping five tracts with zero cases or zero population). Cases and deaths were aggregated into two time periods for analysis: “Phase 1” (March–June 2020) and “Phase 2” (September 2020 to February 2021). The 2020 summer months were a period of reduced COVID‐19 burden of cases/deaths in MA; the exclusion of those months in our modeling allowed comparison of periods of time with similar levels of disease burden.

### 2.2 Identification of institutional outcomes

Using geocoded addresses, we identified institutional cases and deaths among individuals residing in a long‐term care facility (LTCF), prison, or homeless shelter in MA. Locations and capacities of licensed nursing homes, rest homes, and assisted‐living facilities (collectively aggregated to total number of LTCF beds) were obtained from MassGIS.^24^ Likewise, locations of all state, county, and federal correctional facilities in MA were obtained from MassGIS.^25^ Prison data excluded temporary processing or treatment facilities without residential inmates. Locations of homeless shelters in MA were provided by the MDPH Office of Population Health upon request.

### 2.3 Community‐level predictors

We obtained total population counts, as well as select social, occupational, housing, and demographic data at census tract resolution from the most recent five‐year (2015–2019) American Community Survey (ACS).^26^ We evaluated potential predictors hypothesized to be associated with increased risk of disease transmission, disease severity, and/or health disparities, including: population proportions of those who identify as Black or African American, Hispanic or Latino (Latinx), or American Indian or Alaska Native (AIAN); share of population with ages greater than 80 years and share with ages under 20 years; percent of population enrolled as undergraduate students or employed in essential services; the number of LTCF beds per capita; the percent of households with more than 1.5 persons per room; and housing unit density (number of housing units per square mile). We defined “essential services” following the approach of the American Civil Liberties Union (ACLU) Massachusetts.^27^ These variables are informed by and consistent with our previous modeling work at the town level, in which we used backwards model selection to select non‐correlated covariates (confirming that none of the independent variables had a correlation of |*r*| > 0.60).^28^

### 2.4 Statistical analysis

We used mixed‐effects negative binomial regression models to generate incidence rate ratios (IRRs) and 95% confidence intervals for each predictor in the model. We modeled case and death outcomes separately for each Phase, and we fit case and death models inclusive and exclusive of cases/deaths identified as institutional (yielding eight models in total). A random effect of town (351 towns in MA) was included to address within‐town spatial autocorrelation of residuals for nearby tracts. We used counts of cases or deaths at each census tract as the outcome variable, with census tract population used as an offset term to reflect consistent rates. Predictors that affected modeling estimates significantly or that demonstrated changes between the Phases were retained in the models, as were predictors of *a priori* interest to health disparities or specific COVID‐19 risk factors regardless of statistical significance (e.g., housing unit density and proportion of AIAN residents). All statistical analyses were conducted in R (version 4.0.3)^29^ using the “glmmTMB” function from the *glmmTMB* package (version 1.0.2.9).^30^

## 3 RESULTS

Total cases, deaths, and average community characteristics differed between Phase 1 and Phase 2 of the COVID‐19 pandemic in Massachusetts (Table 1). Phase 1 had substantially fewer cases than Phase 2 (99,051 vs. 407,525), but more deaths (7285 vs. 6207). Compared to Phase 1, non‐institutional outcomes in Phase 2 accounted for greater shares of total cases (96.6% vs. 80.1%) and deaths (57.0% vs. 37.0%). Geocoding was highly successfully at matching individuals with census tracts of residence, with each outcome group having at least a 99.7% match rate; in total, 1360 cases (0.27%) were excluded from the models due to inability to geocode to a census tract.

**TABLE 1:** Descriptive statistics by phase of COVID‐19 cases, deaths, and average census tract characteristics in Massachusetts, USA

	Phase 1 (March–June 2020)				Phase 2 (September 2020 to February 2021)			
	Total cases	Non‐inst. cases	Total deaths	Non‐inst. deaths	Total cases	Non‐inst. cases	Total deaths	Non‐inst. deaths
**# of Individuals** ^a^	98 898	79 348	7283	2696	406 318	393 551	6204	3535
**Mean Tract‐Level Characteristics of Strata of Cases/Deaths (Std. Dev.)**								
**% Age < 20**	23.42% (6.05%)	23.87% (6.06%)	22.16% (5.69%)	22.56% (6.06%)	23.32% (5.93%)	23.41% (5.92%)	22.25% (5.44%)	22.79% (5.68%)
**% Age > 80**	4.24% (2.8%)	3.76% (2.4%)	5.86% (3.33%)	4.69% (2.96%)	4.08% (2.53%)	4.02% (2.46%)	5.64% (3.46%)	4.7% (2.94%)
**% AIAN**	0.31% (0.77%)	0.31% (0.79%)	0.24% (0.61%)	0.26% (0.65%)	0.25% (0.71%)	0.25% (0.71%)	0.23% (0.66%)	0.24% (0.67%)
**% Black**	12.32% (17.28%)	13.63% (18.4%)	8.41% (13.34%)	12.13% (17.88%)	8.89% (13.63%)	8.98% (13.77%)	7.06% (11.9%)	8.99% (14.46%)
**% Latinx**	19.88% (21.97%)	21.91% (22.93%)	12.85% (17.35%)	16.17% (19.64%)	16.71% (20.51%)	16.91% (20.68%)	12.07% (16.47%)	14.66% (18.65%)
**% Uninsured**	3.16% (2.6%)	3.4% (2.65%)	2.29% (1.97%)	2.69% (2.16%)	2.82% (2.35%)	2.84% (2.36%)	2.4% (2.03%)	2.64% (2.19%)
**% Essential Workers**	33.76% (7.64%)	34.7% (7.3%)	30.83% (7.55%)	32.5% (7.48%)	33.55% (7.15%)	33.69% (7.05%)	31.9% (7.22%)	33.01% (7.05%)
**LTCF Beds per 100 pop.**	1.61 (2.83)	0.95 (1.98)	3.35 (3.71)	1.28 (2.23)	1.05 (2.08)	0.96 (1.96)	2.69 (3.47)	1.25 (2.28)
**% Undergrads**	5.56% (5.58%)	5.6% (5.51%)	5.42% (5.76%)	5.47% (5.54%)	5.88% (7.77%)	5.93% (7.86%)	5.1% (5.11%)	5.26% (5.25%)
**% Crowding (1.5+/Room)**	0.97% (1.46%)	1.06% (1.52%)	0.67% (1.15%)	0.87% (1.32%)	0.81% (1.32%)	0.82% (1.33%)	0.62% (1.08%)	0.72% (1.17%)
**Housing Density (Units/mi** ^**2**^ **)**	3526 (4112)	3851 (4251)	2476 (3554)	3216 (4262)	2943 (4063)	2996 (4097.3)	2130 (3115)	2657 (3554)

^a^ Reflects the number of cases and deaths that were successfully geocoded to a census tract (total number of individuals excluded due to lack of geocoding is 1360 [0.27%]).

### 3.1 Total cases and deaths

Our first models assessed the relationship between the total number of COVID‐19 cases and deaths in each census tract in Phase 1 and Phase 2 (four models in total). These models included all cases and deaths, including those in institutional settings.

#### 3.1.1 Models predicting total case incidence

**FIGURE 1:**
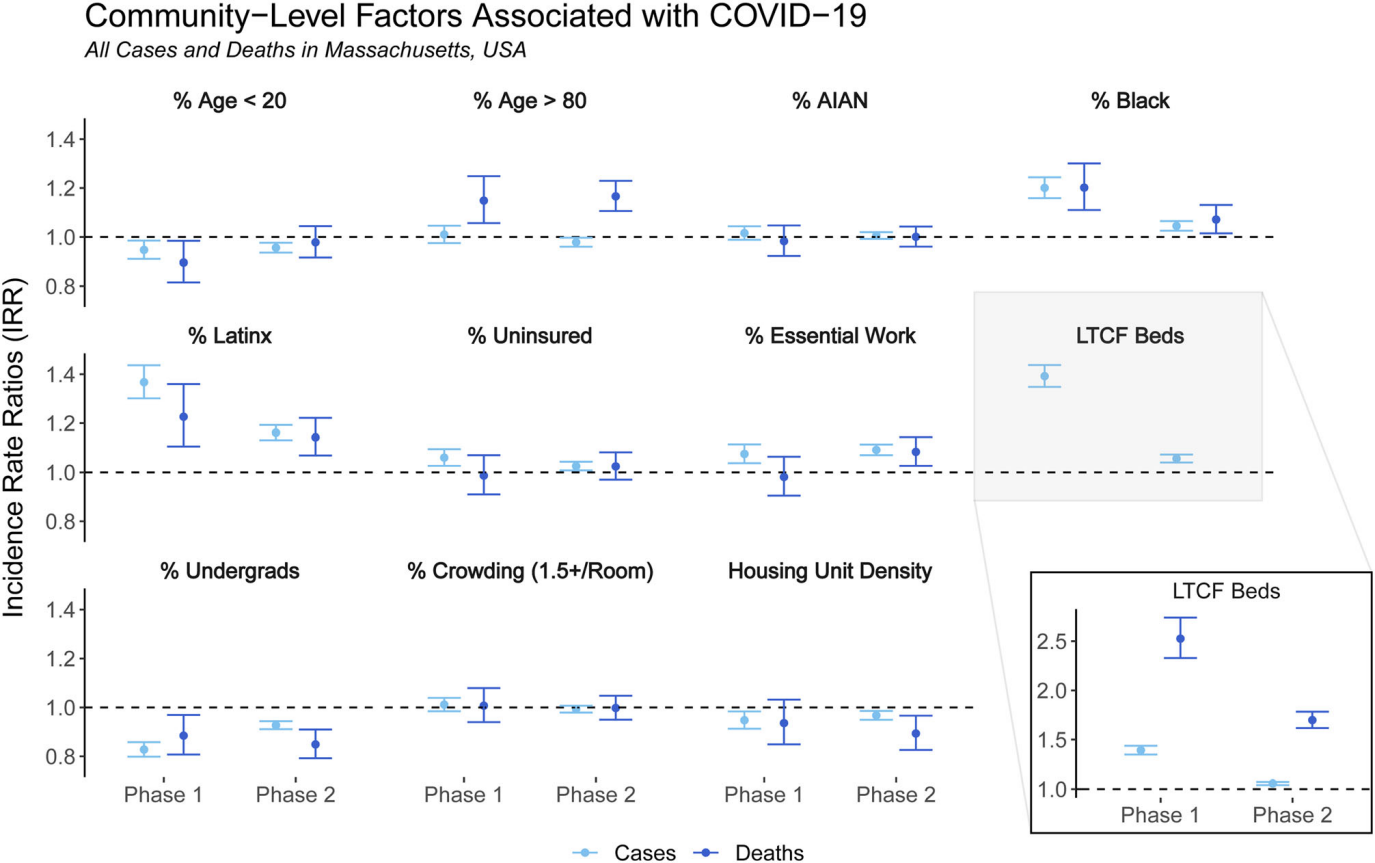
Incidence rate ratios (IRR) for census tract‐level factors included in the regression models for total cases (lighter blue, on left) and total deaths (darker blue, on right) by phase of the pandemic in Massachusetts (Phase 1: March–June 2020, Phase 2: September 2020 to February 2021). Inset for LTCF Beds variable provided to show IRRs for deaths, which are outside the scale of the other variables

Models of key predictors of COVID‐19 *cases* by census tract in each of the two phases are presented in Figure 1 (left points, in lighter blue). Overall, census tract characteristics consistently associated with increased risk of COVID‐19 cases included higher population proportions of essential workers, those without health insurance, Black or Latinx residents, and number of LTCF beds per capita. By contrast, proportion of residents aged 20 years or younger, proportion of undergraduate students, and housing density were significantly associated with *decreased* IRRs. Crowded housing, % aged over 80 years, and proportion of AIAN residents were not statistically significantly associated with tract‐level COVID‐19 case incidence rates. All associations were statistically stable between Phase 1 and Phase 2 (i.e., there were no changes in directionality or statistical significance), but a few variables had substantive attenuations in magnitudes (i.e., more than a 10% change in point estimates toward the null): IRRs for % Black decreased from 1.20 (95% confidence interval [CI]: 1.16, 1.24) in Phase 1 to 1.04 (1.03, 1.06) in Phase 2, % Latinx decreased from 1.37 (1.30, 1.44) to 1.16 (1.13, 1.19), LTCF beds decreased from 1.39 (1.35, 1.44) to 1.06 (1.04, 1.07), and % undergraduates *increased* from 0.83 (0.80, 0.86) to 0.93 (0.91, 0.94).

#### 3.1.2 Models predicting total mortality incidence

Models of key predictors of COVID‐19 *deaths* by census tract in each of the two phases are presented in Figure 1 (right points, in darker blue). Overall, tract‐level characteristics consistently associated with increased risk of COVID‐19 mortality included population proportions of Black or Latinx residents, share of population aged over 80 years, and number of LTCF beds per capita. The only variable consistently associated with decreased risk of census‐tract mortality rates was proportion of undergraduate students. Crowded housing, proportion of AIAN residents, and rate of uninsured individuals were not statistically significantly associated with tract‐level COVID‐19 deaths in either phase. While most variables were statistically stable between Phase 1 and Phase 2 (i.e., consistent directionality and statistical significance), proportion of essential workers went from not statistically significant in Phase 1 (IRR: 0.98 [0.90, 1.06]) to statistically significantly positive in Phase 2 (IRR: 1.08 [1.03, 1.14]), and housing density went from not statistically significant (0.94 [0.85, 1.03]) to statistically significantly negative (0.89 [0.83, 0.97]). Other variables had substantive attenuations in their point estimates between Phase 1 and Phase 2 (i.e., greater than 10% change toward the null): IRRs decreased for proportion of Black residents (decreasing from 1.20 [1.11, 1.30] in Phase 1 to 1.07 [1.01, 1.13] in Phase 2) and LTCF beds per capita (decreasing from 2.53 [2.33, 2.74] to 1.70 [1.62, 1.78]), while IRRs *increased* for proportion of residents aged 20 years or younger, albeit to become statistically non‐significant (increasing from 0.90 [0.82, 0.98] to 0.98 [0.92, 1.04]).

### 3.2 Non‐institutional cases and deaths

#### 3.2.1 Models predicting non‐institutional case incidence

**FIGURE 2:**
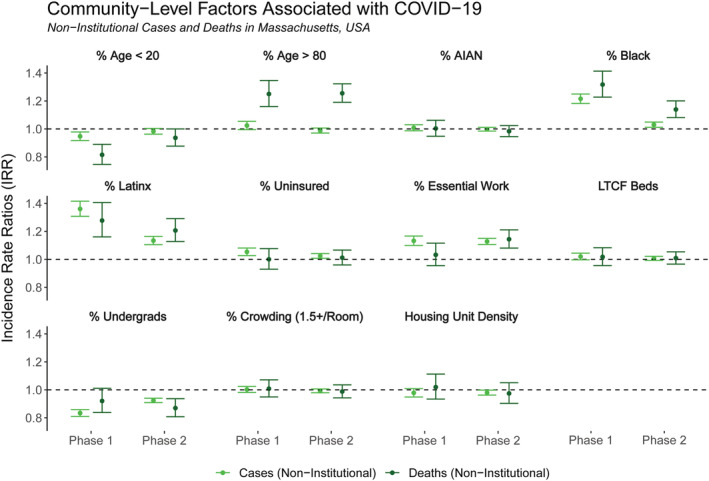
Incidence rate ratios (IRR) for census tract‐level factors included in the regression models for non‐institutional cases (lighter green, on left) and non‐institutional deaths (darker green, on right) by phase of the pandemic in Massachusetts (Phase 1: March–June 2020, Phase 2: September 2020 to February 2021). Non‐institutional cases and deaths are defined as confirmed COVID‐19 cases and deaths geolocated to an address not affiliated with a long‐term care facility, prison, or shelter

Variables associated with non‐institutional cases are shown in Figure 2 (left points in lighter green). As with the models of all cases, consistent, statistically significant positive associations in both Phase 1 and Phase 2 included proportions of Black and Latinx residents, essential services workers, and those without health insurance. Proportion of undergraduate students was the only consistent, statistically significant negative association for non‐institutional cases. Substantive attenuations toward the null between Phases 1 and 2 were exhibited in % Black (decreasing from 1.22 [1.18, 1.25] to 1.03 [1.01, 1.05]), % Latinx (decreasing from 1.36 [1.31, 1.42] to 1.13 [1.11, 1.16]), and % undergraduates (increasing from 0.83 [0.80, 0.86] to 0.93 [0.91, 0.94]). LTCF beds per capita, population proportions of AIAN residents, housing density, and share of individuals aged over 80 years were not statistically significant predictors of non‐institutional cases.

#### 3.2.2 Models predicting non‐institutional death incidence

Variables associated with non‐institutional deaths are shown in Figure 2 (right points in darker green). Consistent, statistically significant positive associations in both Phase 1 and Phase 2 include proportions of Black and Latinx residents, as well as the percent of people aged over 80 years. No covariate was found to have statistically significant negative associations in both phases, though % aged under 20 years was negative in Phase 1 (0.82 [0.75, 0.89]) and % undergraduates was negative in Phase 2 (0.87 [0.81, 0.94]). Substantive changes in point estimates were seen for % of workers in essential services, which increased from 1.03 (0.96, 1.12) in Phase 1 to 1.14 (1.08, 1.21) in Phase 2, and for % Black, which decreased from 1.32 (1.23, 1.41) to 1.14 (1.08, 1.20).

### 3.3 Comparisons between total and non‐institutional cases and deaths

We observed some similarities and differences in predictors between the models for total cases and deaths and for non‐institutional cases and deaths (Table 2). Most notably, the strongly positive associations between LTCF beds per capita and total cases and deaths were sharply reduced to statistical non‐significance in the non‐institutional models, per our original hypotheses. All other variables had confidence intervals that overlapped between the total and non‐institutional models, and estimates were similar across all remaining variables in both phases between total and non‐institutional cases. However, a few substantive differences emerged in the IRR point estimates from models with total versus non‐institutional deaths, albeit the confidence intervals overlapped. Associations between % Black, % Latinx, % aged over 80 years, and % essential workers and non‐institutional deaths were greater than the associations with total deaths across both phases. The largest absolute difference was for the % Black variable in Phase 1 deaths, which was 1.20 (1.11, 1.30) for total deaths and 1.32 (1.23, 1.41) for non‐institutional deaths.

**TABLE 2:** Incidence rate ratios (IRRs) for COVID‐19 cases and deaths in MA census tracts, by total and non‐institutional settings and phase of the pandemic

	COVID‐19 Cases Incidence Rate Ratios (95% Confidence Interval)			
	Phase 1		Phase 2	
Predictors	Total Cases	Non‐Inst. Cases	Total Cases	Non‐Inst. Cases
**% Age < 20**	0.95 (0.91, 0.99)	0.95 (0.92, 0.98)^	0.96 (0.94, 0.98)	0.98 (0.96, 1.00)
**% Age > 80**	1.01 (0.97, 1.05)^	1.02 (1.00, 1.05)^	0.98 (0.96, 1.00)^	0.99 (0.97, 1.01)^
**% AIAN**	1.02 (0.99, 1.04)	1.01 (0.99, 1.03)	1.01 (0.99, 1.02)	1.00 (0.98, 1.01)
**% Black**	1.20 (1.16, 1.24)	1.22 (1.18, 1.25)	1.04 (1.03, 1.06)	1.03 (1.01, 1.05)^
**% Latinx**	1.37 (1.30, 1.44)	1.36 (1.31, 1.42)	1.16 (1.13, 1.19)	1.13 (1.11, 1.16)
**% Uninsured**	1.06 (1.03, 1.09)	1.05 (1.03, 1.08)	1.03 (1.01, 1.04)	1.02 (1.01, 1.04)
**% Essential Workers**	1.08 (1.04, 1.11)	1.13 (1.10, 1.17)	1.09 (1.07, 1.11)	1.13 (1.11, 1.15)
**LTCF Beds per 100 pop.**	**1.39 (1.35, 1.44)^**	**1.02 (1.00, 1.04)**	**1.06 (1.04, 1.07)^**	**1.01 (0.99, 1.02)**
**% Undergrads**	0.83 (0.80, 0.86)	0.83 (0.81, 0.86)	0.93 (0.91, 0.94)	0.92 (0.91, 0.94)
**% Crowding (1.5+/Room)**	1.01 (0.98, 1.04)	1.00 (0.98, 1.02)	0.99 (0.98, 1.01)	0.99 (0.98, 1.01)
**Housing Density (Units/mi^2^)**	0.95 (0.91, 0.99)	0.95 (0.92, 0.98)	0.96 (0.94, 0.98)	0.98 (0.96, 1.00)

	COVID‐19 Deaths Incidence Rate Ratios (95% Confidence Interval)			
	Phase 1		Phase 2	
Predictors	Total Deaths	Non‐Inst. Deaths	Total Deaths	Non‐Inst. Deaths
**% Age < 20**	0.90 (0.82, 0.98)	0.82 (0.75, 0.89)^	0.98 (0.92, 1.04)	0.94 (0.88, 1.00)
**% Age > 80**	1.15 (1.06, 1.25)^	1.25 (1.16, 1.35)^	1.17 (1.11, 1.23)^	1.26 (1.19, 1.32)^
**% AIAN**	0.98 (0.92, 1.05)	1.00 (0.95, 1.06)	1.00 (0.96, 1.04)	0.98 (0.95, 1.02)
**% Black**	1.20 (1.11, 1.30)	1.32 (1.23, 1.41)	1.07 (1.01, 1.13)	1.14 (1.08, 1.20)^
**% Latinx**	1.23 (1.11, 1.36)	1.28 (1.16, 1.41)	1.14 (1.07, 1.22)	1.21 (1.13, 1.29)
**% Uninsured**	0.99 (0.91, 1.07)	1.00 (0.93, 1.08)	1.02 (0.97, 1.08)	1.01 (0.96, 1.07)
**% Essential**	0.98 (0.90, 1.06)	1.03 (0.96, 1.12)	1.08 (1.03, 1.14)	1.14 (1.08, 1.21)
**LTCF Beds**	**2.53 (2.33, 2.74)^**	**1.02 (0.96, 1.08)**	**1.70 (1.62, 1.78)^**	**1.01 (0.97, 1.05)**
**% Undergrads**	0.88 (0.81, 0.97)	0.92 (0.84, 1.01)	0.85 (0.79, 0.91)	0.87 (0.81, 0.94)
**% Crowding**	1.01 (0.94, 1.08)	1.01 (0.95, 1.07)	1.00 (0.95, 1.05)	0.99 (0.94, 1.04)
**Housing Density**	0.94 (0.85, 1.03)	1.02 (0.93, 1.11)	0.89 (0.83, 0.97)	0.97 (0.90, 1.05)

*Note*: Bold values indicate non‐overlapping confidence intervals between total and non‐institutional outcomes (rows); carets (^) indicate non‐overlapping confidence intervals between cases and deaths within the same type of outcome (columns).

## 4 DISCUSSION

By using individual geocoded addresses of COVID‐19 cases and deaths in MA, we were able to both evaluate community‐level risk for these outcomes at high spatial resolution and distinguish institutional outcomes from those in community models of disease over time. Removing institutional cases from our models, especially in the context of mortality endpoints where institutional facilities contributed an appreciable percentage of total deaths, allowed for a more nuanced understanding of local risk and disease drivers. Additionally, assessing trends over time across both case and mortality outcomes shed light on differential case fatality by subpopulation over time. Overall, our efforts highlight the value of collaboration between state public health departments and academic researchers to access, analyze, and interpret COVID‐19 data to maximize its effective use in public health practice.

We observed key disparities in models of both cases and death outcomes associated with the proportion of Black and Latinx populations by tract, findings that parallel those at town‐level resolution across a shorter time period, as well as findings from other studies.^28^, ^31^, ^32^, ^33^, ^34^ The variability observed in the size of these estimates over time reinforces how these race and ethnicity variables reflect social constructs and not biological or constant risk factors. Broader assessments of the structural factors that result in these disparities ‐ notably systemic racism and its consequences associated with inequities in wealth, healthcare, housing, and employment ‐ are vital to comprehensively understand why these communities have experienced elevated COVID‐19 risk. The variable IRRs over time could be coupled with other community‐level information to better assess the particular factors driving disparities in each phase of the pandemic.

The finding that the association between disease incidence and % Black residents was smaller during Phase 2 than Phase 1 may indicate that public health policies and other measures enacted by Fall 2020 among communities with higher proportions of Black residents successfully reduced risk relative to other communities. This could include greater availability of testing compared to availability during the early months of the pandemic. Notably, we did not see as substantial of a reduction for communities with higher proportions of Latinx residents, which points toward the need for a closer look at testing as well as structural barriers to implementing risk‐reduction methods (such as ability to self‐isolate, work from home, or physically distance) across MA communities.

Our study period captured only the early months of vaccination availability in the state, reflecting availability for healthcare workers (beginning on December 15, 2020), residents of long‐term care facilities (December 28, 2020), and all individuals aged 75 and over (February 1, 2021). It is possible that our findings related to racial/ethnic patterns for Phase 2 reflect, in part, differential vaccination patterns from early 2021; however, this is unlikely to have a large influence on our overall findings, given the limited number of individuals fully vaccinated by the end of the time period assessed here. Given that LTCF residents were eligible for vaccination for several weeks of the study period, it is plausible that our estimate for the LTCF beds per capita variable is an underestimate of the true association.

Case risk associated with proportion essential workers by tract remained elevated across both time periods in our study. This finding may reflect the limitations of workplace interventions to reduce exposure risk among this workforce, including challenges to physical distancing in essential service jobs, and lack of paid sick leave.^35^ Our study period did not overlap with vaccine availability targeting essential workers outside of healthcare settings (March 22, 2021); analyses utilizing more recent data could consider the effect of targeted vaccination on these estimates by tract. The elevated mortality risk observed in association with proportion essential workers by tract, especially in Phase 2, may suggest that communities with more essential workers faced a higher case fatality rate, although it is difficult to conclude with certainty from our data.

One important aspect of modeling cases and deaths separately across pandemic phases is the ability to effectively control for differential testing availability and utilization. This was particularly salient during Phase 2, which saw increased availability of testing to the public and widespread asymptomatic surveillance testing at workplaces and schools. Since testing is not uniformly distributed across all census tracts,^36^ IRRs are likely higher in places with more testing, irrespective of case severity. By contrast, COVID‐19 deaths in Phase 2 are expected to have much more consistent identification, making comparisons less susceptible to testing biases. The greater mortality risk for Black/Latinx populations, essential workers, and older residents that we observed in Phase 2 may thus provide a more accurate depiction of racial and ethnic inequities in COVID‐19 impacts than case rates alone, which could potentially be more useful for informing targeted public health interventions.

By retaining LTCF facilities in non‐institutional models, we attempted to estimate the impact of LTCF cases on disease incidence outside of these facilities. Our data indicate that density of long‐term care services was not a major correlate of infections or deaths in the community at large (among individuals who were not residing in these institutions; see Section 3.2). This observation may indicate successful biocontainment within these facilities or limited interaction between residents/employees and community members. However, we lack key information to definitively make this determination, including information on controls within facilities and residential location of LTCF employees. Our findings also may indicate that risk factors for institutional cases and deaths differed in meaningful ways from what was observed at the community level. While beyond the scope of this study, considerations of risk factors for mortality specifically within institutions, and comparisons between these within‐institution and community‐level risk factors, would be a valuable addition to this literature.

Our analysis is limited by a few key factors. First, census tracts are heterogenous and not distinctly classified by the variables in our models, which complicates the interpretation of our findings. We identified tracts using continuous demographic and occupational characteristics, and the same town may have elevated proportions of some, but not all, of the covariates in our model. As such, our analyses can serve as a guide for understanding differential risk by population subgroups but not to identify specific tracts to target with public health interventions, as would be possible with spatial methods. Additionally, as mentioned previously, limited availability of testing during Phase 1 resulted in testing and diagnosis of only symptomatic cases early in the pandemic, while testing was widely available in Phase 2, both for symptomatic cases and asymptomatic surveillance. It is difficult to concretely assess the directionality of these biases, but these trends may indicate that our data from Phase 1 reflects underestimates of true associations. In addition, the variables we included might not be all, or the strongest, predictors of cases or deaths. Another limitation is that patient address information likely contains errors, which cascade into the geocoding process, resulting in misclassification of LTCF residents and tract of residence; however, this is likely non‐differential with respect to outcome and, moreover, there is no feasible way to ameliorate this type of error. Our age‐related covariates may imprecisely classify risk associated with age. Finally, ACS data were derived pre‐pandemic and may not fully reflect conditions during the pandemic, especially with respect to employment and housing.

Our project is strengthened by our geocoded individual‐level data, which was facilitated by a cross‐sectoral partnership. Using address geocodes of individual patients who were diagnosed or died of COVID‐19, we observed significant disparities in both case and mortality burden in association with population proportions of Black and Latinx residents, as well as essential workers at the census tract level in the first year of the pandemic in Massachusetts. Modeling cases and deaths separately, as well as with and without institutional outcomes, allowed us to more comprehensively understand health disparities experienced by vulnerable subgroups during the first year of the pandemic.

## AUTHOR CONTRIBUTIONS

**Keith Spangler:** Formal analysis; methodology. **Prasad Patil:** Conceptualization; formal analysis; methodology; supervision. **Xiaojing Peng:** Formal analysis; methodology. **Jonathan Levy:** Conceptualization; funding acquisition; supervision. **Kevin Lane:** Conceptualization; funding acquisition; methodology; resources; supervision. **Koen Tieskens:** Formal analysis; methodology. **Fei Carnes:** Data curation; methodology; resources. **R. Monina Klevens:** Methodology; resources. **Elizabeth Erdman:** Methodology; resources. **T. Troppy:** Methodology; resources. **M. Fabian:** Conceptualization; funding acquisition. **Jessica Leibler:** Conceptualization; formal analysis; funding acquisition; methodology; supervision.

### PEER REVIEW

The peer review history for this article is available at https://publons.com/publon/10.1111/irv.12926.

## Data Availability

The community‐level covariate data from the American Community Survey and other public sources are available from the corresponding author upon reasonable request. The COVID‐19 outcome data were made available to the authors by the Massachusetts Department of Public Health under a data‐use agreement and cannot be shared by the authors.
